# Jeffrey’s insights: Jeffrey Modell Foundation’s global genetic sequencing pilot program to identify specific primary immunodeficiency defects to optimize disease management and treatment

**DOI:** 10.1007/s12026-020-09131-x

**Published:** 2020-05-27

**Authors:** Jessica Quinn, Vicki Modell, Jennifer Holle, Rebecca Truty, Swaroop Aradhya, Britt Johnson, Jordan Orange, Fred Modell

**Affiliations:** 1 Jeffrey Modell Foundation, 780 Third Avenue, 47th Floor, New York City, NY 10017 USA; 2 Invitae, San Francisco, CA USA

**Keywords:** Genetic sequencing, Gene sequencing, Sequencing, Next generation sequencing (NGS), Primary immunodeficiency (PI), Jeffrey Modell Foundation (JMF), Jeffrey Modell Centers Network (JMCN)

## Abstract

Primary immunodeficiencies (PI) are genetic defects of the immune system that result in chronic and often life-threatening infections and/or life-threatening autoimmunity if not diagnosed and treated. Patients with a suspected PI, but without a genetic diagnosis, commonly undergo a diagnostic odyssey that is costly, time-consuming, and arduous. This delay in diagnosis prevents appropriate disease management and treatment, contributing to prolonged suffering and decreased quality of life. Although next generation sequencing (NGS) can provide these patients with relief from such a diagnostic odyssey, it is often unavailable, mainly due to cost and inaccessibility. In January 2019, the Jeffrey Modell Foundation (JMF) launched a free genetic sequencing pilot program for Jeffrey Modell Centers Network (JMCN) patients clinically diagnosed with an underlying PI. A total of 21 sites within the JMCN were invited to participate. JMF collaborated with Invitae, and testing was comprised of Invitae’s Primary Immunodeficiency Panel, which currently includes 207 genes. A questionnaire was disseminated to each participating physician to evaluate barriers to access to genetic sequencing and changes in disease management and treatment after testing. One hundred fifty-eight patients and 29 family members were tested in this pilot study. Twenty-one percent of patients with a suspected monogenic disorder received a molecular diagnosis, and others received potentially useful diagnostic leads. Based on the results of genetic sequencing, clinical diagnosis was altered in 45% of patients, disease management was altered in 40%, treatment was altered in 36%, and genetic counseling was altered in 62%. The results of this pilot program demonstrate the utility, cost-efficiency, and critical importance of NGS for PI and make the case for broad scale sequence–based diagnostics for PI patients when requested by expert immunologists.

**Electronic supplementary material:**

The online version of this article (10.1007/s12026-020-09131-x) contains supplementary material, which is available to authorized users.

## Introduction

### Primary immunodeficiency

Primary immunodeficiencies (PI) [1, 2] are genetic disorders of the immune system that result in chronic, serious, and often life-threatening infections, and/or life-threatening autoimmunity if not diagnosed and treated [3, 4]. There are over 400 genetically defined single-gene inborn errors of immunity [5, 6]. In addition to diseases as serious as severe combined immunodeficiency (SCID), manifestations of less severe PIs may include susceptibility to common infections, opportunistic infections, persistent or aberrant inflammation, and severe organ-specific autoimmune conditions.

Recent studies have shown that PI may be more common than previously estimated [7] and that as much as 1% of the population may be affected with a PI when all types and varieties are considered [8]. Recently, improvements in molecular diagnosis, whole exome sequencing, and insight from innovative treatments have led to a better understanding of the immune system, as well as, improved quality of life for those living with PI [9–13]. However, awareness of PI among physicians and the general public remains challenging, and there continues to be a need for improved and timely management of these conditions [14, 15].

Patients without a genetic diagnosis commonly undergo a diagnostic odyssey including numerous specialist referrals and an exhaustive number of expensive and often unhelpful tests [16]. Delays in diagnosis, and therefore disease management and treatment, contribute to continuing suffering by the patient, with chronic, recurring infections and in some cases, organ or tissue damage, or even death. Finally, the expense borne by health care systems and even the patients themselves owing to diagnostic odysseys is not to be underestimated.

### Next generation sequencing

Rapid technological developments in next generation sequencing (NGS) have provided relief in many cases from the diagnostic odyssey. NGS allows for fast and less costly sequencing of DNA and RNA by allowing many genes to be sequenced simultaneously, revolutionizing the approach to rare disease. Many PIs share overlapping clinical presentations, so diagnostic NGS gene panels or whole exome sequencing can facilitate rapid diagnosis by addressing differential diagnoses.

A genetic etiology for PI is prevalent among patients who fulfill clinical diagnostic criteria for the individual PI diagnoses. Each of the clinical categories has numerous genetic etiologies that can individually serve as prognostic indicators of disease severity and can influence treatment decisions. It is therefore vital to investigate the genetic underpinnings of PI to the fullest extent available [9]. While this has historically fallen upon research laboratories, the introduction of high fidelity diagnostic NGS and exome sequencing has brought definitive diagnosis into broader reach.

The molecular diagnostic rate of NGS has been found to range from 15 to 46%, with a median rate of 25%, in a systematic review of eight studies using NGS in a mixed PI population [17]. There is precedent in genetic testing leading to a change in diagnosis and management of PI disease. Outcomes from NGS have significantly influenced patient diagnosis and management. One study in otherwise difficult to diagnose PI patients documented an alteration of clinical diagnoses in 55% of the immunodeficient patients who had sequencing findings and a change to clinical management in 25% [9]. NGS and related platforms are quickly becoming recognized as a vital part of the clinical care of patients with a suspected PI. It also appears that early application of these approaches may result in benefits to the patient while providing value to health care systems.

Presently, these valuable resources are frequently unavailable due to perceived cost and insurance constraints, accessibility challenges, and difficulty with interpretation, with cost being the most frequently reported and burdensome barrier [18]. In fact, it has been reported that the most common clinical testing denied by insurers is genetic testing [18]. In addition, many clinics and insurers require referrals to a geneticist before genetic testing may be conducted, despite the expert qualification of clinical immunologists to evaluate the need for such testing [18]. As a result, there are myriad patients that have genetically definable PI who have not been evaluated or who have endured unnecessary expense in receiving evaluation.

### Jeffrey Modell Centers Network

Jeffrey Modell Foundation (JMF) established a network of specialized centers over the past decade, the Jeffrey Modell Centers Network (JMCN), to provide the necessary infrastructure for referral, earliest possible diagnosis, appropriate treatments, and cutting-edge research. Currently, the JMCN consists of 821 expert physicians at 379 institutions, in 294 cities, and 86 countries spanning six continents, and continues to expand. Approximately one-third of the JMCN is in the US, with 116 centers in the US, and 263 OUS. There are over 187,000 patients followed in the JMCN, but the majority have not received a genetic diagnosis [19].

JMF offers the unique advantage of utilizing existing sequencing technologies but applying an extraordinary level of pre-test probability by leveraging the vast expertise within the JMCN, which provides organized and direct access to the most expert immunologists, a majority of whom harbor numerous clinically diagnosed patients in need of genetic diagnostics. Through the utilization of the JMCN, we have the ability to link those patients most likely to have a genetic diagnosis to genetic diagnostics. By harnessing the expertise of clinical immunologists within the JMCN, we demonstrate that their clinical index of suspicion alone should equate with the rationale for NGS testing.

## The pilot program

In January 2019, JMF launched a genetic sequencing pilot program for patients clinically diagnosed with an underlying PI disease. The aim of this initiative was to help identify a specific genetic cause and provide medical professionals a precise diagnosis to hopefully either confirm or advance appropriate management and treatment. Through this pilot program, we sought to demonstrate the value and clinical utility of NGS for PI through JMF’s unique and established network, which we hypothesized would provide a high level of pre-test probability. JMF offered this program to the community as a free service. No hospitals, patients, physicians, insurance companies, or government agencies were charged.

An additional aim of this pilot program included determining the rate of revision of clinical diagnosis as well as any change in disease management enabled by genetic diagnosis or lack thereof. These outcomes were able to be obtained through our ongoing relationship with the expert immunologists in the JMCN. Overall, we hypothesized that revision of diagnosis and change in disease management of the patients would both occur at a meaningful rate.

Given the advantage of direct access to JMF’s network of experts in identifying patients with the greatest need, we predicted the molecular diagnostic rate resulting from this study to be within the 15–46% range reported in the recently published systematic review of NGS in PI [17]. We hypothesized that when such probability exists from an expert immunologist (based upon their clinical index of suspicion), no other testing or rationale is needed to achieve a diagnostic rate within this range and that there would be a 25% disease management alteration rate. We also hypothesize that this and related initiatives will help define genetic testing as the “first line” intervention when expert immunologists have a high pre-test probability for a genetic PI.

In addition to resulting in improved patient outcomes, a revision of diagnosis and management would be substantially cost saving, both in the short- and long-term. Through this pilot study, we also aimed to evaluate cost-efficiency and importance of genetic testing from a health services perspective, the rationale for broad scale sequence–based diagnostics for PI and to potentially justify greater access to NGS sequencing in the right context.

## Methods

A total of 21 sites within the JMCN were invited to participate in the pilot program and contribute their highest pre-test probability patients, for a total of up to 160 patients enrolled across all sites. There were no criteria applied, and the samples to submit were solely at the discretion of the JMCN immunologist. Approximately 50% of participating sites were located in the US, with 10 sites in the US and 11 sites outside the US including Cairo, Egypt; Lima, Peru; Nablus, Palestine; San Jose, Costa Rica; Medellin, Colombia; Prague, Czech Republic; two sites in Santiago, Chile; Barcelona, Spain; Quito, Ecuador; and Mexico City, Mexico.

### Description of testing services

JMF collaborated with Invitae, a fully certified clinical diagnostic laboratory which performs full-gene sequencing and intragenic deletion-duplication analysis using NGS technology. Testing was comprised of Invitae’s Primary Immunodeficiency Panel, which currently includes 207 genes (Table S1). The participating center provided samples to Invitae according to Invitae’s instructions set forth in their online portal.

NGS testing was performed by Invitae as previously described [20], and variant interpretation was carried out based on an expansion of the American College of Medical Genetics guidelines [21]. Patient results were reviewed and categorized by variant classifications, i.e., negative (no reportable variants were identified), positive (pathogenic or likely pathogenic (P/LP) variants identified), and uncertain (variants of uncertain significance (VUS) identified). Of note, increased risk alleles (common variants associated with an elevated risk, but not a diagnosis, of a disorder) were excluded from analysis as these variants are very common in the general population. Positive results were further categorized by their clinical relevance (i.e., carrier status, molecular diagnosis, see Table 1).

**Table 1:** Clinical relevance of genetic test results

Confirmed or likely molecular diagnosis	Carrier status	Heterozygous results
1 heterozygous P/LP allele in AD gene	1 heterozygous allele in AR gene	1 heterozygous allele in a gene with AR and AD inheritance
1 hemizygous P/LP allele in XL gene in male	1 heterozygous allele in XL gene in female	
2 heterozygous or 1 homozygous P/LP alleles in AR genes		
1 heterozygous P/LP allele and 1 VUS in AR genes		

### Patient eligibility

This pilot program included those patients that participating physicians suspected as having a PI and identified as being among the most severe and compelling, but that had not yet received a genetic diagnosis. Patient eligibility was based upon the highest pre-test probability of the highly qualified expert clinicians at participating JMCN centers. Appropriateness was confirmed by JMF scientific and medical staff, and the rationale was shared with the Invitae research team. Testing was ordered by a clinician at a JMF center participating in this program. One of the following clinical indications was recommended: (i) confirmed clinical diagnosis of PI, (ii) newborn screen suggestive of PI, (iii) suspected clinical diagnosis of PI, following the JMF 10 Warning Signs of PI (http://www.info4pi.org/library/educational-materials/10-warning-signs).

### Protocol

Each patient that participated in this pilot program completed a consent form, which was signed in person by both the physician and the patient or guardian before sample collection. The patient then provided a specimen sample at the participating JMF center. Invitae accepted blood, saliva, and extracted genomic DNA sample types. The specimen sample and consent form were sent to Invitae for genetic sequencing. Invitae performed full-gene sequencing and exon level deletion/duplication analysis using NGS technology on all specimen samples. The turn-around time was 10–21 days with a 14-day average. Reports were provided that included interpretation of the identified variant(s) and were made available to the ordering clinician through Invitae’s online portal. Family variant testing was made available at no additional charge for any proband that was found to have a likely pathogenic or pathogenic variant in this pilot.

### The JMF Questionnaire

A brief questionnaire was developed and disseminated to each participating physician to collect data to evaluate barriers to access and changes in disease management and treatment for each patient sequenced through this program (Figure S1). Any and all information provided on this questionnaire was de-identified. Obtaining this information was in an effort to understand impact and importance of genetic sequencing for patients with a suspected PI.

## Results

### Genetic sequencing results

One hundred fifty-eight patients and 29 family members were tested in this pilot study. At least one reportable genetic variant (interpreted to be either pathogenic, likely pathogenic, or of uncertain significance) was identified in 151 of 158 samples received and sequenced (96%), for a total of 507 genetic variants identified, as the majority of patients (75%) had multiple reportable variants identified. Fifty-six P/LP variants were identified in 52 patients (33%). Copy number variants (CNV) comprised 5% of P/LP variants identified. Table 2 displays all variants identified by the International Union of Immunological Societies (IUIS) category (2017 classification).

**Table 2:** Variants by IUIS category

IUIS category	Number of variants
Antibody deficiencies	35
Autoinflammatory deficiencies	67
Combined T/B cell deficiencies	107
Disorders of immune dysregulation	86
Diseases of intrinsic and innate immunity	62
Immunodeficiencies affecting cellular and humoral immunity	97
Phagocytic defects	34
Other defects	19
Total	507

**Fig. 1:**
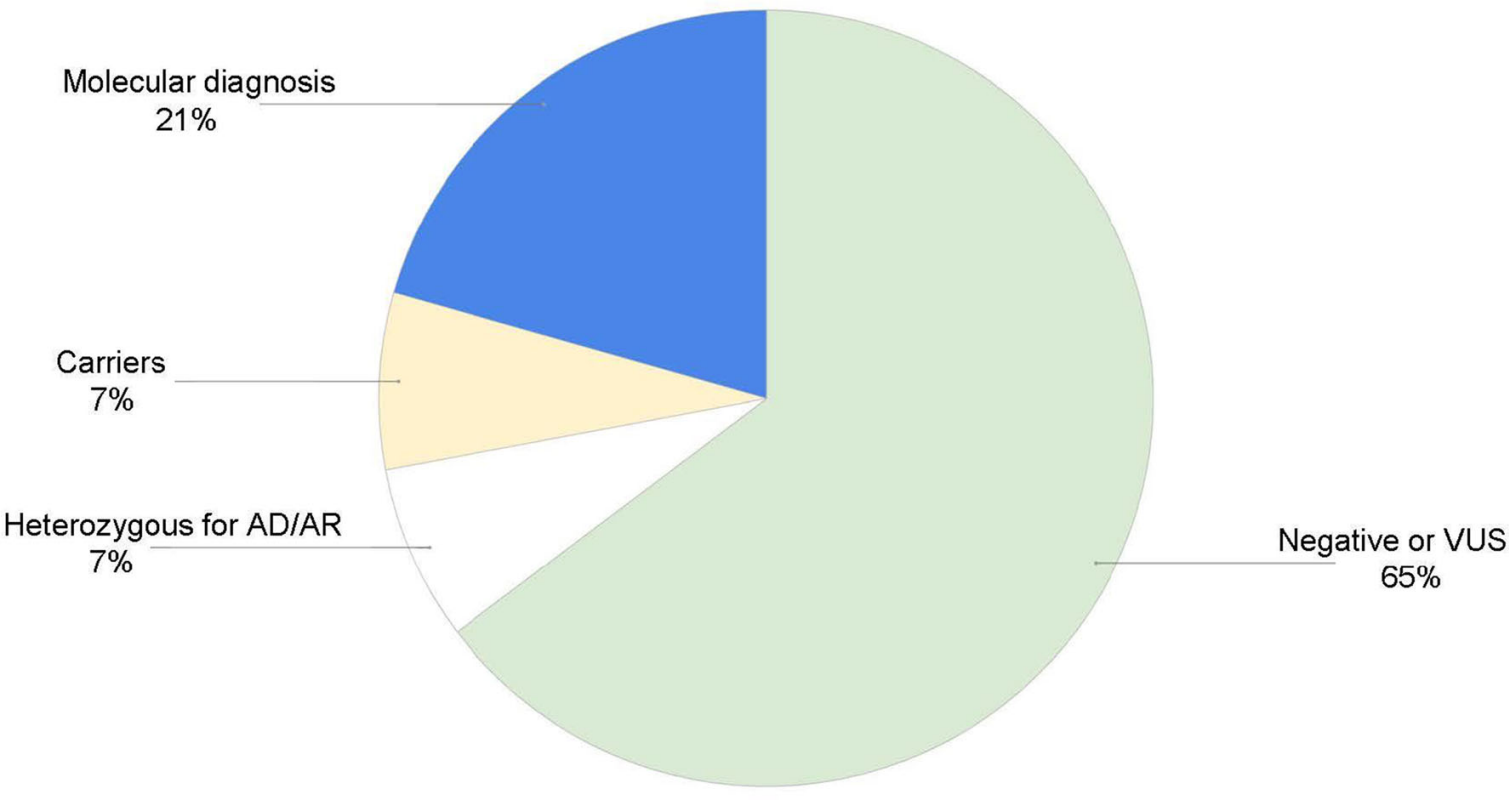
Clinical results

Patients’ indications for genetic testing were broad, however 22 patients were diagnosed with, or suspected to have, common variable immunodeficiency (CVID). CVID is rarely due to a monogenic cause and is more often multifactorial in nature [22]. None of the patients referred for CVID received a molecular diagnosis from this panel, though one patient was found to be a heterozygous carrier of a TACI pathogenic mutation, which, while alone is not diagnostic, is considered a risk factor for CVID. Excluding the patients with CVID, 136 patients underwent genetic testing for suspected monogenic disorders, and 28 (21%) received a molecular diagnosis (Fig. 1). In addition, 10 patients (7%) were found to be heterozygous carriers of autosomal recessive conditions, and 10 patients (7%) were heterozygous for variants in genes with autosomal recessive (AR) and autosomal dominant (AD) inheritance patterns, in which the positive finding may or may not explain the patient’s phenotype. The most common molecular diagnoses are shown in Table 3, categorized according to the IUIS Expert Committee classification of inborn errors of immunity.

**Table 3:** Molecular diagnoses by gene

IUIS category	Condition	Gene	Number of patients	Genotypes
Antibody deficiencies	X-linked agammaglobulinemia	BTK	4	c.1631+5G>C (Intronic)
				c.1901G>C (p.Trp634Ser)
				c.496C>T (p.Gln166*)
				c.179_181del (p.Lys60del)
	X-linked hyper-IgM syndrome	CD40LG	2	c.474del (p.Lys159Asnfs*3)
				c.761C>T (p.Thr254Met)
Autoinflammatory deficiencies	Polyarteritis nodosa	ADA2	2	c.1085G>A (p.Trp362*) (hom)
				c.973-2A>G (Splice acceptor); c.934C>T (p.Arg312*)
CID with associated or syndromic features	Ataxia-telangiectasia	ATM	2	c.4019_4029del (p.Leu1340Cysfs*10); c.2817del (p.Lys940Asnfs*9)
				c.3802del (p.Val1268*); c.2921+1G>A (Splice donor)
	CHARGE syndrome	CHD7	1	c.4944_4945del (p.Tyr1649Leufs*3)
	Immunodeficiency-centromeric instability-facial anomalies syndrome	DNMT3B	1	c.1838T>C (p.Val613Ala); c.2292G>T (p.Arg764Ser)
	Hepatic veno-occlusive disease with immunodeficiency	SP110	1	c.686dup (p.Gln231Profs*5) (hom)
Disorders of immune dysregulation	Hermansky-Pudlak syndrome	AP3B1	1	c.779G>A (p.Trp260*) (hom)
	LRBA deficiency	LRBA	2	c.3286_3287del (p.Phe1096Leufs*3) (hom)
				Deletion (Exon 35) (hom)
	X-linked lymphoproliferative disease	XIAP	1	c.664C>T (p.Arg222*)
Diseases of intrinsic and innate immunity	STAT1 gain-of-function; mycobacterial disease	STAT1	1	c.820C>T (p.Arg274Trp)
Immunodeficiencies affecting cellular and humoral immunity	Adenosine deaminase deficiency	ADA	1	c.218+1G>A (Splice donor) (hom)
	Severe combined immunodeficiency	NHEJ1	2	c.178-1G>A (Splice acceptor) (hom)
				c.178-1G>A (Splice acceptor) (hom)
	Severe combined immunodeficiency	RAG1	2	c.2275C>T (p.Arg759Cys); c.1228C>T (p.Arg410Trp)
				c.322C>T (p.Arg108*); c.1835A>G (p.His612Arg)
	Severe combined immunodeficiency	RAG2	1	c.686G>A (p.Arg229Gln) (hom)
	Severe combined immunodeficiency	ZAP70	1	c.261C>G (p.Tyr87*) (hom)
Phagocytic defects	Chronic granulomatous disease	CYBB	1	c.742dup (p.Ile248Asnfs*36)
	Neutropenia	ELANE	2	c.607G>C (p.Gly203Arg)
				c.597+1G>A (Splice donor)

The average age among patients tested was 13 years. The average age among patients receiving a genetic diagnosis was 7.5 years with 93% tested during the pediatric period. The diagnostic yield among children aged 0–5 years was trending highest, with 28% of patients receiving a molecular diagnosis. Fourteen percent of patients in the 6–17 years age group received a molecular diagnosis compared with only 9% of patients in the 18 and older group. Interestingly, when patients tested for indications of CVID were excluded, the diagnostic yield in the adult group was 17%, indicating that when probable multifactorial causes are not included, the likelihood of finding a genetic etiology in these patients is also substantial. These figures and patient demographics are listed in Table 4.

**Table 4:** Patient demographics

	Number of patients (%)	Patients positive (diagnostic rate, %)
Gender		
Male	99 (63)	18 (18)
Female	59 (37)	10 (17)
Total	158 (100)	
Age		
<5	57 (36)	16 (28)
6–17	66 (40)	9 (14)
18 +	35 (22)	3 (9)
Total	158 (100)	

### The JMF Questionnaire results

As part of this pilot program, physicians were asked to fill out a questionnaire to gain more insights into the participating patients’ clinical care, hospitalizations, estimated healthcare costs, and changes to clinical management post-testing (Figure S1). Of the 158 participating patients, we received results for 119 (75%).

#### Prior to genetic testing

Physicians reported that in the past 12 months, participating patients saw a health care provider an average of 5.28 times, 42% of the participating patients had been admitted to the hospital at least once, 34% of the participating patients had visited an emergency room at least once, and 13% of the participating patients had been admitted to the ICU at least once. It is important to note that some patients had multiple admissions. It was reported that 8.5% of the participating patients did not seek care for the condition because of cost, and 72% of the participating patients did not seek genetic testing because of cost. The average annual estimated cost of care, medication, and treatment prior to genetic sequencing was $75,699. In the United States, only 3% of participating patients have insurance coverage for genetic testing. Outside of the United States, 37% of participating patients have coverage for genetic testing.

#### After genetic testing

Responding physicians reported a suspicion of a particular diagnosis in 75% of the patients. However, after genetic testing, respondents altered their suspected diagnosis in 67% of these patients. Based on the results of genetic sequencing, clinical diagnosis was altered in 45% of all patients, disease management was altered in 40% of all patients, treatment was altered in 36% of all patients, and genetic counseling was altered in 62% of all patients. Other at-risk or affected individuals were identified in the family for 14% of the patients. Importantly, based on the results of genetic sequencing, 45% of the patients had a change in outcomes, and there is an applicable therapy for 80% of the diagnosed patients.

### Costs of care

We leveraged our healthcare economic experience in PI [19, 23] to attach health care dollar values to the most frequent conditions affecting PI patients (Table 5) [24–32]. The results of the JMF Questionnaire demonstrate that these costs would be reduced with appropriate disease management and treatment facilitated by an accurate diagnosis accessed through genetic sequencing.

**Table 5:** Costs of the most frequent conditions affecting patients with PI

Condition	Average no. of episodes	Cost per episode	Annual cost
Persistent otitis media	4.2	$528	$2217
Serious sinus and upper respiratory infections	4.6	$1125	$5175
Viral infections	3.7	$1275	$4717
Acute bronchitis	3.1	$1700	$5270
Bacterial pneumonias	2.8	$3552	$9945
Bronchiectasis	4.3	$3165	$13,609
Hospitalization days	19.8	$2480	$49,104
Physician/ER visits	70.8	$180	$12,744
Days on antibiotics	166.2	$10	$1662
School/work days missed	33.9	$195	$6610
Total per patient			$111,053

## Discussion

Patients with a suspected PI, but without a genetic diagnosis, commonly undergo a diagnostic odyssey that is costly, slow, time-consuming, and arduous. This delay in diagnosis can prevent the patient from receiving appropriate disease management and treatment, contributing to prolonged suffering, expense, and decreased quality of life. Although NGS can provide these patients with knowledge, hope, and relief from such a diagnostic odyssey, it is often unavailable, mainly due to cost and inaccessibility.

Through this pilot program, we demonstrated the value and clinical utility of NGS for PI through JMF’s unique and established network. By leveraging the vast JMCN, which provides direct access to expert immunologists who harbor a backlog of clinically diagnosed patients in need of a genetic diagnosis, we demonstrated that their clinical expertise alone proves to be very effective in determining the need for NGS. We specifically did not apply restrictive criteria for which patients could or could not be sequenced through this program, believing simply in the value of when an expert immunologist feels a patient with PI should be sequenced.

In this pilot, the diagnostic rate was found to be 21%, excluding CVID patients. This falls within the expected range of 15–46%, as reported in a systematic review of eight studies using NGS in a mixed PI population [17]. The median diagnostic rate in this systemic review was found to be 25%, with four studies ranging from 15 to 25% and four studies ranging from 40 to 46%. It is important to note specific features of each study that reported diagnostic rates of 40–46% including the use of whole exome sequencing (WES) in one study [9], a greater number of genes on the test panel in one study [33], testing of a highly consanguineous population in one study [34], and inclusion of patients that already had at least one known causal mutation in one study [35].

Additionally, at the 2018 American Society of Human Genetics meeting, Invitae presented an internal diagnostic rate for their PI panel of 7% [36]. We postulate that the higher diagnostic rate of 21% found in this pilot study is due to the high pre-test probability gained through the expertise of the ordering immunologist. This clearly illustrates the diagnostic acumen and expertise of the ordering immunologist in identifying patients who are truly in need of a genetic diagnosis.

Notably, in this pilot, 33% of the identified variants were reported as P/LP. However, it was reported through the JMF Questionnaire that after genetic sequencing, clinical diagnosis was altered in 45% of patients, disease management was altered in 40% of patients, treatment was altered in 36% of patients, and genetic counseling was altered in 62% of patients. This indicates that clinicians are able to use non-P/LP results in order to “rule out” specific PIs, which can still impact the medical management of their patients. This is valuable information as it demonstrates the importance and utility of sequencing in disease management, even when no official diagnosis is received.

Through the JMF Questionnaire we also established that patients, both in the US and OUS, faced barriers to obtaining genetic sequencing such as lack of insurance coverage, prohibitive cost, and limited access. The cost and burden of frequent hospital admissions, emergency room visits, and ICU admissions also proved to be a major challenge for these patients. In addition to improved patient outcomes, a revision of diagnosis and management due to genetic sequencing would result in substantial cost savings, both in the short- and long-term, and generate the case for immediately moving to PI NGS testing when an expert immunologist has high pre-test probability for a genetic disease. To demonstrate this point, we leveraged our healthcare economic experience in PI [19, 23] to attach health care dollar values to the most frequent conditions affecting PI patients (Table 5) [24–32], which would be considerably reduced with appropriate disease management and treatment facilitated by an accurate diagnosis that could be accessed through genetic sequencing. These reduced costs, furthermore, do not account for the use of additional expensive phenotypic or immunologic tests which are likely to be necessary without a definitive genetic diagnosis. Interestingly, many of these tests do not face the same insurance, access, or coverage obstacles that genetic tests do. The cost-saving potential of genetic sequencing for PI patients alone should serve as a mandate for broad scale sequence–based diagnostics for PI and justify greater access to NGS sequencing in the right context.

## Conclusion

The results of this pilot program demonstrate the utility, cost-efficiency, and critical importance of NGS for PI, and make the case for broad scale sequence–based diagnostics for PI patients when requested by expert immunologists, given the high pre-test probability. Many physicians have a backlog of high priority patients that do not have access to genetic sequencing, as demonstrated by this program. Physician specialists should be able to access genetic tests when warranted. Indeed, the experts are right when it comes to a need for genetic tests. We envision a future, in which any government or private health agency will be compelled to support genetic testing for PI as an initial intervention when a clinical diagnosis has been established and pre-test probability is confirmed by an expert immunologist.

## Electronic supplementary material

ESM 1 (DOCX 256 kb)

## Abbreviations

JMF: Jeffrey Modell Foundation
JMCN: Jeffrey Modell Centers Network
PI: Primary immunodeficiency
NGS: Next generation sequencing
SCID: Severe combined immunodeficiency
CVID: Common variable immune deficiency
AR: Autosomal recessive
AD: Autosomal dominant
XL: X-linked
DNA: Deoxyribonucleic acid
RNA: Ribonucleic acid
US: United States
OUS: Outside of the United States
VUS: Variants of uncertain significance
P/LP: Pathogenic or likely pathogenic
CNV: Copy number variant
IUIS: International Union of Immunological Societies
WES: Whole exome sequencing

## References

[1] Modell V (2007). The impact of physician education and public awareness on early diagnosis of primary immunodeficiencies. Immunol Res.

[2] Modell F (2007). Immunology today and new discoveries: building upon legacies of Dr. Robert A. Good. Immunol Res.

[3] Cunningham-Rundles C, Ponda PP (2005). Molecular defects in T- and B-cell primary immunodeficiency diseases. Nat Rev Immunol.

[4] Cooper MA, Pommering TL, Koranyi K (2003). Primary immunodeficiencies. Am Fam Physician.

[5] BousfihaA JL, Al-HerzW AF, Casanova JL, Chatila T (2015). The 2015 IUIS phenotypic classification for primary immunodeficiencies. J Clin Immunol.

[6] Tangye SG, Al-Herz W, Bousfiha A (2020). Human inborn errors of immunity: 2019 update on the classification from the International Union of Immunological Societies Expert Committee. J Clin Immunol.

[7] Bousfiha AA, Jeddane L, Ailal F, Benhsaien I, Mahlaoui N, Casanova JL, Abel L (2013). Primary immunodeficiency diseases worldwide: more common than generally thought. J Clin Immunol.

[8] Boyle JM, Buckley RH (2007). Population prevalence of diagnosed primary immunodeficiency diseases in the United States. J Clin Immunol.

[9] Stray-Pedersen A, Sorte HS, Samarakoon P, Gambin T, Chinn IK, Coban Akdemir ZH, Erichsen HC, Forbes LR, Gu S, Yuan B, Jhangiani SN, Muzny DM, Rødningen OK, Sheng Y, Nicholas SK, Noroski LM, Seeborg FO, Davis CM, Canter DL, Mace EM, Vece TJ, Allen CE, Abhyankar HA, Boone PM, Beck CR, Wiszniewski W, Fevang B, Aukrust P, Tjønnfjord GE, Gedde-Dahl T, Hjorth-Hansen H, Dybedal I, Nordøy I, Jørgensen SF, Abrahamsen TG, Øverland T, Bechensteen AG, Skogen V, Osnes LTN, Kulseth MA, Prescott TE, Rustad CF, Heimdal KR, Belmont JW, Rider NL, Chinen J, Cao TN, Smith EA, Caldirola MS, Bezrodnik L, Lugo Reyes SO, Espinosa Rosales FJ, Guerrero-Cursaru ND, Pedroza LA, Poli CM, Franco JL, Trujillo Vargas CM, Aldave Becerra JC, Wright N, Issekutz TB, Issekutz AC, Abbott J, Caldwell JW, Bayer DK, Chan AY, Aiuti A, Cancrini C, Holmberg E, West C, Burstedt M, Karaca E, Yesil G, Artac H, Bayram Y, Atik MM, Eldomery MK, Ehlayel MS, Jolles S, Flatø B, Bertuch AA, Hanson IC, Zhang VW, Wong LJ, Hu J, Walkiewicz M, Yang Y, Eng CM, Boerwinkle E, Gibbs RA, Shearer WT, Lyle R, Orange JS, Lupski JR (2017). Primary immunodeficiency diseases: genomic approaches delineate heterogeneous Mendelian disorders. J Allergy Clin Immunol.

[10] Rider NL, Kutac C, Hajjar J, Scalchunes C, Seeborg FO, Boyle M, Orange JS (2017). Health-related quality of life in adult patients with common variable immunodeficiency disorders and impact of treatment. J Clin Immunol.

[11] Quinti I, Di Pietro C, Martini H, Pesce AM, Lombardi F, Baumghartner M (2012). Health related quality of life in common variable immunodeficiency. Yonsei Med J.

[12] Casanova JL, Abel L, Quintana-Murci L (2013). Immunology taught by human genetics. Cold Spring Harb Symp Quant Biol.

[13] Meyts I, Bosch B, Bolze A, Boisson B, Itan Y, Belkadi A, Pedergnana V, Moens L, Picard C, Cobat A, Bossuyt X, Abel L, Casanova JL (2016). Exome and genome sequencing for inborn errors of immunity. J Allergy Clin Immunol.

[14] Modell V, Gee B, Lewis DB, Orange JS, Roifman CM, Routes JM, Sorensen RU, Notarangelo LD, Modell F (2011). Global study of primary immunodeficiency diseases (PI)—diagnosis, treatment, and economic impact: an updated report from the Jeffrey Modell Foundation. Immunol Res.

[15] Modell F, Puente D, Modell V (2009). From genotype to phenotype. Further studies measuring the impact of a physician education and public awareness campaign on early diagnosis and management of primary immunodeficiencies. Immunol Res.

[16] Sawyer SL, Hartley T, Dyment DA, Beaulieu CL, Schwartzentruber J, Smith A, Bedford HM, Bernard G, Bernier FP, Brais B, Bulman DE, Warman Chardon J, Chitayat D, Deladoëy J, Fernandez BA, Frosk P, Geraghty MT, Gerull B, Gibson W, Gow RM, Graham GE, Green JS, Heon E, Horvath G, Innes AM, Jabado N, Kim RH, Koenekoop RK, Khan A, Lehmann OJ, Mendoza-Londono R, Michaud JL, Nikkel SM, Penney LS, Polychronakos C, Richer J, Rouleau GA, Samuels ME, Siu VM, Suchowersky O, Tarnopolsky MA, Yoon G, Zahir FR, Majewski J, Boycott KM, FORGE Canada Consortium, Care4Rare Canada Consortium (2016). Utility of whole-exome sequencing for those near the end of the diagnostic odyssey: time to address gaps in care. Clin Genet.

[17] Yska HAF, Elsink K, Kuijpers TW, Frederix GWJ, van Gijn ME, van Montfrans JM (2019). Diagnostic yield of next generation sequencing in genetically undiagnosed patients with primary immunodeficiencies: a systematic review. J Clin Immunol.

[18] Heimall JR, Hagin D, Hajjar J, Henrickson SE, Hernandez-Trujillo HS, Tan Y, Kobrynski L, Paris K, Torgerson TR, Verbsky JW, Wasserman RL, Hsieh EWY, Blessing JJ, Chou JS, Lawrence MG, Marsh RA, Rosenzweig SD, Orange JS, Abraham RS (2018). Use of genetic testing for primary immunodeficiency patients. J Clin Immunol.

[19] Modell V, Orange JS, Quinn J, Modell F (2018). Global report on primary immunodeficiencies: 2018 update from the Jeffrey Modell Centers Network on disease classification, regional trends, treatment modalities, and physician reported outcomes. Immunol Res.

[20] Lincoln SE, Kobayashi Y, Anderson MJ, et al. A systematic comparison of traditional and multigene panel testing for hereditary breast and ovarian cancer genes in more than 1000 patients. J Mol Diagn. 2015;17(5):533–44. 10.1016/j.jmoldx.2015.04.009. 10.1016/j.jmoldx.2015.04.009 26207792

[21] Nykamp K, Anderson M, Powers M (2020). Correction: Sherloc: a comprehensive refinement of the ACMG-AMP variant classification criteria. Genet Med.

[22] Bogaert DJA, Dullaers M, Lambrecht BN, Vermaelen KY, de Baere E, Haerynck F (2016). Genes associated with common variable immunodeficiency: one diagnosis to rule them all?. J Med Genet.

[23] Modell V, Quinn J, Ginsberg G, Gladue R, Orange J, Modell F (2017). Modeling strategy to identify patients with primary immunodeficiency utilizing risk management and outcome measurement. Immunol Res.

[24] Agency for Healthcare Research and Quality. http://www.hcup.ahrq.gov 2015. 10.1080/15360280802537332 21923316

[25] Aetna Member Navigator. http://www.aetna.com 2015.

[26] Centers for Medicare and Medicaid Services. Berenson-eggers type of service (BETOS). 2015. https://www.cms.gov/Medicare/Coding/HCPCSReleaseCodeSets/BETOS.html.

[27] Centers for Medicare and Medicaid Services. Medicare current beneficiary survey (MCBS). 2015. https://www.cms.gov/Research-Statistics-Data-and-Systems/Research/MCBS/index.html?redirect=/mcbs.

[28] Congressional Budget Office. Factors underlying the growth in Medicare’s spending for physicians’ services. 2007. Retrieved 28 Apr 2010, from http://www.cbo.gov/ftpdocs/81xx/doc8193/06-06-MedicareSpending.pdf.

[29] Bundorf KM, Royalty A, Baker LC (2009). Health care cost growth among the privately insured. Health Aff.

[30] TruvenHealth Analytics Healthcare spending index for employer-sponsored insurance. 2014.

[31] Fronstin P. Sources of health insurance and characteristics of the uninsured: analysis of the March 2013 current population survey, vol 390. EBRI (Employee Benefit Research Institute) Issue Brief No. 390, 2013, Washington, DC; 2013. p. 1–36. 24195154

[32] Health Care Cost Institute (HCCI) 2014 Health Care Cost and Utilization Report 2015. http://www.healthcostinstitute.org/files/2014%20HCCUR%2010.29.15.pdf.

[33] Rae W, Ward D, Mattocks C, Pengelly RJ, Eren E, Patel SV, Faust SN, Hunt D, Williams AP (2018). Clinical efficacy of a next-generation sequencing gene panelfor primary immunodeficiency diagnostics. Clin Genet.

[34] Bisgin A, Boga I, Yilmaz M, Bingol G, Altintas D (2018). The utility of next-generation sequencing for primary immunodeficiency disorders: experience from a clinical diagnostic laboratory. Biomed Res Int.

[35] Moens LN, Falk-Sorqvist E, Asplund AC, Bernatowska E, Smith CIE (2014). Diagnostics of primary immunodeficiency diseases: a sequencing capture approach. PLoS One.

[36] Nicolosi P, Holle J, Truty R, Yu H, Hartshorne C, Martin S, Johnson B (2018). Expanded genetic testing for primary immunodeficiencies: findings from a 207-gene next-generation sequencing panel.

