# Supplementary material for: Jeffrey’s insights: Jeffrey Modell Foundation’s global genetic sequencing pilot program to identify specific primary immunodeficiency defects to optimize disease management and treatment
Source: Immunol Res. 2020 May 27;68(3):126–34. doi: 10.1007/s12026-020-09131-x (PMC7335369; doi:10.1007/s12026-020-09131-x)

**SUPPLEMENTAL MATERIAL**

**Table S1. Genes included on Invitae PID panel**

| **Gene** | **Disorder** |
| --- | --- |
| ACD | AD/AR-DKC due to TPP1 deficiency |
| ACP5 | Spondyloenchondro-dysplasia with immune dysregulation |
| ACTB | β-actin deficiency |
| ADA | Adenosine deaminase (ADA) deficiency |
| ADA2 | ADA2 deficiency |
| ADAM17 | ADAM17 deletion |
| ADAR | ADAR1 deficiency, Aicardi-Goutieres syndrome 6 |
| AICDA | AID deficiency |
| AIRE | APECED (APS-1), autoimmune polyendocrinopathy with candidiasis and ectodermal dystrophy |
| AK2 | Reticular dysgenesis, AK2 deficiency |
| AP3B1 | Hermansky-Pudlak syndrome, type 2 |
| ATM | Ataxia-telangiectasia |
| B2M | MHC class I deficiency |
| BCL10 | BCL10 deficiency |
| BLNK | BLNK deficiency |
| BLOC1S6 | Hermansky-Pudlak syndrome, type 9 |
| BTK | BTK deficiency |
| CARD11 | CARD11 deficiency, CARD11 gain of function |
| CARD14 | CAMPS (CARD14 mediated psoriasis) |
| CARD9 | CARD9 deficiency |
| CASP10 | ALPS-Caspase 10 |
| CASP8 | ALPS IIb, caspase 8 deficiency |
| CD247 | CD3ζ deficiency |
| CD27 | CD27 deficiency |
| CD3D | CD3δ deficiency |
| CD3E | CD3ε deficiency |
| CD3G | CD3γ deficiency |
| CD40LG | CD40 ligand deficiency |
| CD79A | Igα deficiency |
| CD79B | Igβ deficiency |
| CD8A | CD8 deficiency |
| CEBPE | Specific granule deficiency |
| CHD7 | CHARGE syndrome |
| CIITA | MHC class II deficiency |
| CLPB | 3-Methylglutaconic aciduria |
| COPA | COPA defect, autoimmune interstitial lung, joint, and kidney disease (AILJK) |
| CORO1A | Coronin-1A deficiency |
| CR2 | CD21 deficiency |
| CSF2RA | Pulmonary alveolar proteinosis |
| CSF3R | G-CSF receptor deficiency |
| CTC1 | AR-DKC due to CTC1 deficiency |
| CTLA4 | CTLA4 deficiency (ALPSV) |
| CTPS1 | CTPS1 deficiency |
| CTSC | Papillon-Lefèvre Syndrome |
| CXCR4 | WHIM (warts, hypogammaglo- bulinemia, infections, myelokathexis) syndrome |
| CYBA | Autosomal recessive CGD |
| CYBB | X-linked chronic granulomatous disease (CGD) |
| DCLRE1B | AR-DKC due to DCLRE1B deficiency |
| DCLRE1C | DCLRE1C (Artemis) deficiency |
| DKC1 | XL-DKC due to Dyskerin deficiency |
| DNMT3B | Immunodeficiency with centromeric instability and facial anomalies (ICF1) |
| DOCK2 | DOCK2 deficiency |
| DOCK8 | DOCK8 deficiency |
| ELANE | Elastase deficiency (SCN1), cyclic neutropenia |
| EPG5 | Vici syndrome due to EPG5 deficiency |
| FADD | FADD deficiency |
| FAS | ALPS-FAS |
| FASLG | ALPS-FASLG |
| FERMT3 | Leukocyte adhesion deficiency type 3 |
| FOXN1 | Winged helix deficiency (nude) AAB: syndromic SCID |
| FOXP3 | IPEX, immune dysregulation, polyendocrinopathy, enteropathy X-linked |
| FPR1 | Localized juvenile periodontitis |
| G6PC3 | G6PC3 deficiency (SCN4) |
| GATA2 | GATA2 deficiency |
| GFI1 | GFI 1 deficiency (SCN2) |
| HAX1 | Kostmann Disease (SCN3) |
| ICOS | ICOS deficiency |
| IFIH1 | Aicardi-Goutieres syndrome 7 |
| IFNGR1 | IFN-γ receptor 1 deficiency |
| IFNGR2 | IFN-γ receptor 2 deficiency |
| IGLL1 | λ5 deficiency |
| IKBKB | IKBKB deficiency |
| IL10 | IL-10 deficiency |
| IL10RA | IL-10Rα deficiency |
| IL10RB | IL-10Rβ deficiency |
| IL12B | IL-12p40 deficiency |
| IL12RB1 | IL-12 and IL-23 receptor β1 chain deficiency |
| IL17F | IL-17F deficiency |
| IL17RA | IL-17RA deficiency |
| IL17RC | IL-17RC deficiency |
| IL1RN | IL1RN |
| IL21 | IL-21 deficiency |
| IL21R | IL-21R deficiency |
| IL2RA | CD25 deficiency |
| IL2RG | γc deficiency |
| IL36RN | DITRA – Deficiency of IL-36 receptor antagonist |
| IL7R | IL7Rα deficiency |
| IRAK4 | IRAK-4 deficiency |
| IRF7 | IRF7 deficiency |
| IRF8 | IRF8 deficiency |
| ISG15 | ISG15 deficiency |
| ITCH | ITCH deficiency |
| ITGB2 | Leukocyte adhesion deficiency type 1 |
| ITK | lymphoproliferative syndrome type 1 (LPFS1) |
| JAGN1 | JAGN1 deficiency |
| JAK3 | JAK3 deficiency |
| LAMTOR2 | P14/LAMTOR2 deficiency |
| LCK | LCK deficiency |
| LIG4 | DNA ligase IV deficiency |
| LPIN2 | Chronic recurrent multifocal osteomyelitis and congenital dyserythropoietic anemia (Majeed syndrome) |
| LRBA | LRBA deficiency |
| LYST | Chediak-Higashi syndrome |
| MAGT1 | X-linked immunodeficiency with magnesium defect, Epstein-Barr virus infection, and neoplasia (XMEN) |
| MALT1 | MALT1 deficiency |
| MAP3K14 | NIK deficiency |
| MEFV | Familial Mediterranean Fever |
| MOGS | MOGS deficiency |
| MVK | Mevalonate kinase deficiency |
| MYD88 | MyD88 deficiency |
| NBN | Nijmegen breakage syndrome |
| NCF2 | Autosomal recessive CGD |
| NCF4 | Autosomal recessive CGD |
| NFAT5 | NFAT5 haploinsufficiency |
| NFKB2 | NFKB2 deficiency |
| NFKBIA | Anhidrotic ectodermal dysplasia with T-cell immunodeficiency (EDA-ID), IKBA gain |
| NHEJ1 | Cernunnos/XLF deficiency |
| NHP2 | AR-DKC due to nucleolar protein family A member 2 (NHP2) deficiency |
| NLRC4 | NLRC4-MAS (macrophage activating syndrome), Familial cold autoinflammatory syndrome 4 |
| NLRP12 | Familial cold autoinflammatory syndrome 2 |
| NLRP3 | Muckle-Wells syndrome, Familial cold autoinflammatory syndrome 1, Neonatal onset multisystem inflammatory disease (NOMID) or chronic infantile neurologic cutaneous and articular syndrome (CINCA) |
| NOD2 | Blau syndrome |
| NOP10 | AR-DKC due to nucleolar protein family A member 3 (NHP3) or NOP10 deficiency |
| ORAI1 | ORAI-I deficiency |
| PARN | AR-DKC due to PARN deficiency |
| PGM3 | PGM3 deficiency |
| PIK3CD | Activated PI3K-δ |
| PIK3R1 | PI3KR1 deficiency, PI3KR1 loss of function |
| PLCG2 | PLAID (PLCγ2 associated antibody deficiency and immune dysregulation), Familial cold autoinflammatory syndrome 3, APLAID (autoinflammation and PLCγ2 associated antibody deficiency and immune dysregulation) |
| PMM2 | PMM2-congenital disorder of glycosylation (CDG-Ia) |
| PNP | Purine nucleoside phosphorylase (PNP) deficiency |
| POLE | FILS syndrome |
| PRF1 | Perforin deficiency (FHL2) |
| PRKCD | PRKC delta deficiency |
| PRKDC | DNA PKcs deficiency |
| PSMB8 | CANDLE (chronic atypical neutrophilic dermatitis with lipodystrophy) |
| PSTPIP1 | Pyogenic sterile arthritis, pyoderma gangrenosum, acne (PAPA) syndrome |
| PTPRC | CD45 deficiency |
| RAB27A | Griscelli syndrome, type 2 |
| RAC2 | Rac 2 deficiency |
| RAG1 | RAG 1 deficiency |
| RAG2 | RAG 2 deficiency |
| RBCK1 | Polyglucosan body myopathy, early-onset, with or without immunodeficiency (PBMEI) |
| RFX5 | MHC class II deficiency group C |
| RFXANK | MHC class II deficiency group B |
| RFXAP | MHC class II deficiency group D |
| RHOH | RhoH deficiency |
| RMRP | Cartilage hair hypoplasia |
| RNASEH2A | RNASEH2A deficiency, Aicardi-Goutieres syndrome 4 |
| RNASEH2B | RNASEH2B deficiency, Aicardi-Goutieres syndrome 2 |
| RNASEH2C | RNASEH2C deficiency, Aicardi-Goutieres syndrome 3 |
| RORC | RORc deficiency |
| RTEL1 | AR-DKC due to regulator of telomere elongation (RTEL1) deficiency |
| SAMHD1 | SAMHD1 deficiency, Aicardi-Goutieres syndrome 5 |
| SEMA3E | CHARGE syndrome |
| SH2D1A | SH2D1A deficiency (XLP1) |
| SH3BP2 | Cherubism |
| SLC29A3 | SLC29A3 mutation |
| SLC35C1 | Leukocyte adhesion deficiency type 2 |
| SLC37A4 | Glycogen storage disease type 1b |
| SLC7A7 | lysinuric protein intolerance |
| SMARCAL1 | Schimke Immunoosseous Dysplasia |
| SP110 | Hepatic veno-occlusive disease with immunodeficiency |
| SPINK5 | Comel-Netherton syndrome |
| STAT1 | STAT1 deficiency |
| STAT2 | STAT2 deficiency |
| STAT3 | AD-HIES (Job or Buckley Syndrome), STAT3 GOF mutations |
| STAT5B | STAT5b deficiency |
| STIM1 | STIM1 deficiency |
| STK4 | MST1 deficiency |
| STX11 | Syntaxin 11 deficiency, (FHL4) |
| STXBP2 | STXBP2 / Munc18-2 deficiency (FHL5) |
| TAP1 | MHC class I deficiency |
| TAP2 | MHC class I deficiency |
| TAPBP | MHC class I deficiency |
| TBX1 | DiGeorge syndrome |
| TAZ | Barth syndrome |
| TBX1 | DiGeorge syndrome |
| TCN2 | Transcobalamin 2 deficiency |
| TERC | AD-DKC due to TERC deficiency |
| TERT | AD-DKC due to TERT deficiency |
| TICAM1 | TRIF deficiency |
| TINF2 | AD-DKC due to TINF2 deficiency |
| TLR3 | TLR3 deficiency |
| TMC6 | EVER1 deficiency |
| TMC8 | EVER2 deficiency |
| TMEM173 | STING–associated vasculopathy, infantile onset |
| TNFRSF13B | TACI deficiency |
| TNFRSF13C | BAFF receptor deficiency |
| TNFRSF1A | TNF receptor-associated periodic syndrome (TRAPS) |
| TNFRSF4 | OX40 deficiency |
| TNFSF12 | TWEAK deficiency |
| TPP2 | Tripeptidyl-Peptidase II Deficiency |
| TRAF3 | TRAF3 deficiency |
| TRAF3IP2 | ACT1 deficiency |
| TREX1 | TREX1 deficiency, Aicardi-Goutieres syndrome |
| TRNT1 | TRNT1 deficiency |
| TTC7A | Immunodeficiency with multiple intestinal atresias |
| TYK2 | Tyk2 deficiency |
| UNC13D | UNC13D / Munc13-4 deficiency (FHL3) |
| UNC93B1 | UNC93B1 deficiency |
| UNG | UNG deficiency |
| VPS13B | Cohen syndrome |
| VPS45 | VPS45 deficiency (SCN5) |
| WAS | Wiskott-Aldrich syndrome, X-linked neutropenia/ myelodysplasia |
| WIPF1 | WIP deficiency |
| XIAP | XIAP deficiency (XLP2) |
| ZAP70 | ZAP-70 deficiency |
| ZBTB24 | Immunodeficiency with centromeric instability and facial anomalies (ICF2) |

**Figure S1. JMF Physician Questionnaire**


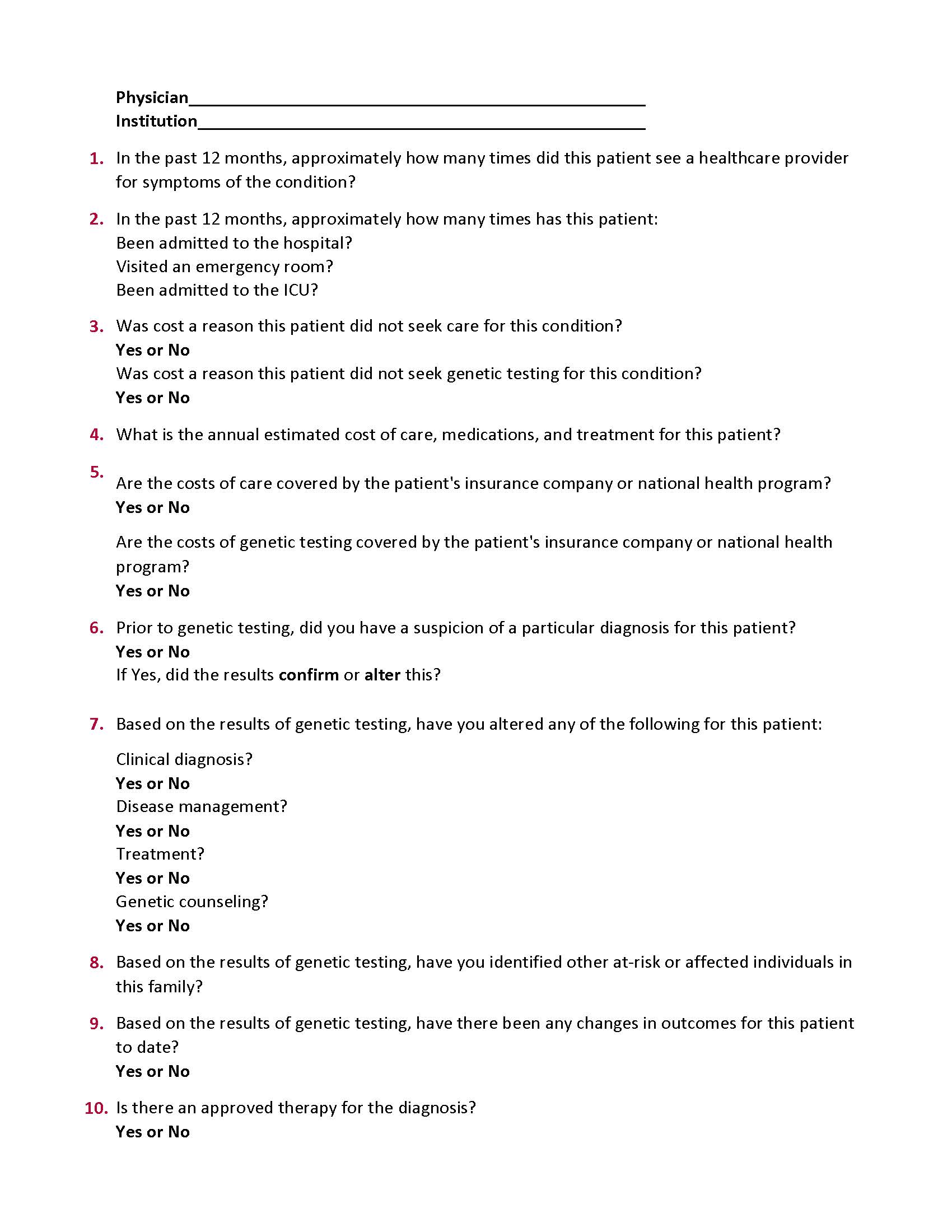

Supplement: Supplementary file 1 — (DOCX 256 kb) [file 12026_2020_9131_MOESM1_ESM.docx]
